# Aneurysmal Bone Cyst of the Head of the Fibula: An Unusual Presentation

**DOI:** 10.7759/cureus.30376

**Published:** 2022-10-17

**Authors:** Radha A Channawar, Sanjay V Deshpande, Sandeep Shrivastav, Swapnil V Date, Hitendra Wamborikar

**Affiliations:** 1 Department of Orthopedics and Traumatology, Jawaharlal Nehru Medical College, Datta Meghe Institute of Medical Sciences, Wardha, IND

**Keywords:** swelling, pain, fibula, aneurysmal bone cyst, bone cyst

## Abstract

Bone cysts are tumor-like lesions of bone. These are primarily of two types: simple or unicameral and aneurysmal bone cysts (ABC). An aneurysmal bone cyst (ABC) is a blood-filled cyst that usually occurs over the metaphysis of long bones, flat bones, and vertebrae. The symptoms of these cysts include pain and swelling over the area. It may consist of pathological fractures as well. The cyst is benign but may invade local tissue and erode bone. The investigations required are radiological and histopathological examinations that further confirm the diagnosis. The differential diagnosis includes giant cell tumor and telangiectatic osteosarcoma. Here, we discuss a case of an aneurysmal bone cyst of the head of the fibula, which is a rare site for ABC to occur (common sites are metaphyseal ends of the femur, humerus, tibia, scapula, and vertebrae). The treatment modalities have a wide range of options that range from en bloc resection to minimally invasive techniques such as selective artery embolization, sclerotherapy, and radiotherapy.

## Introduction

Bone cysts are of two types: simple (unicameral) and aneurysmal bone cysts (ABC). Simple cysts are also called unicameral cysts. These bone cysts are tumor-like pathologies of bone that are characterized by fluid-filled, cystic benign lesions of the long bone. Unicameral cysts are usually seen on the proximal end of long bones such as the humerus and femur. These cystic lesions are generally found in pediatric and adolescent populations (commonly in the second decade of life) [1,2]. An aneurysmal bone cyst is one such highly vascular, osteolytic, locally invasive, benign pathology of the bone that might further lead to multiple pathological fractures. Predominantly, aneurysmal bone cysts are found at the metaphyseal ends of long bones such as the femur, tibia, and spine [1,3-5]. The majority of cases are asymptomatic, which, on further progression of the disease, may present as localized swelling, pain, and sometimes pathological fractures [1,4]. The pathology can be confirmed on radiological investigations, and depending on its site and progression, management can be devised [1,3-5].

## Case presentation

Appropriate consent was taken.

History

A 24-year-old male presented with a single swelling over the lateral aspect of the right knee just beneath the lateral joint line for six months associated with a dull aching type of pain. The pain had increased in the last 10 days. The pain was insidious in onset and was gradually progressive. The pain aggravated on flexion of the knee and relieved with medications and rest. There was no history of falls or trauma. There was no tingling, numbness, or weakness distal to swelling, especially in the ankle and foot.

Examination of swelling

It was a single swelling approximately 3 × 3 cm in size, oval in shape, at the proximal end (head) of the right fibula. The surface was smooth and regular. On palpation, the local temperature was normal, with non-tender swelling with a smooth surface. It was non-fluctuant and incompressible and yielded on pressure. It was not fixed to the skin, subcutaneous tissue, or muscles.

Dorsalis pedis artery and posterior tibial artery pulsations were palpable. There was no sensory or motor deficit in the right ankle and foot. Based on the examination, the following differential diagnoses can be inferred: giant cell tumor of the fibular head, unicameral bone cyst, and aneurysmal bone cyst.

Investigations

**Figure 1:**
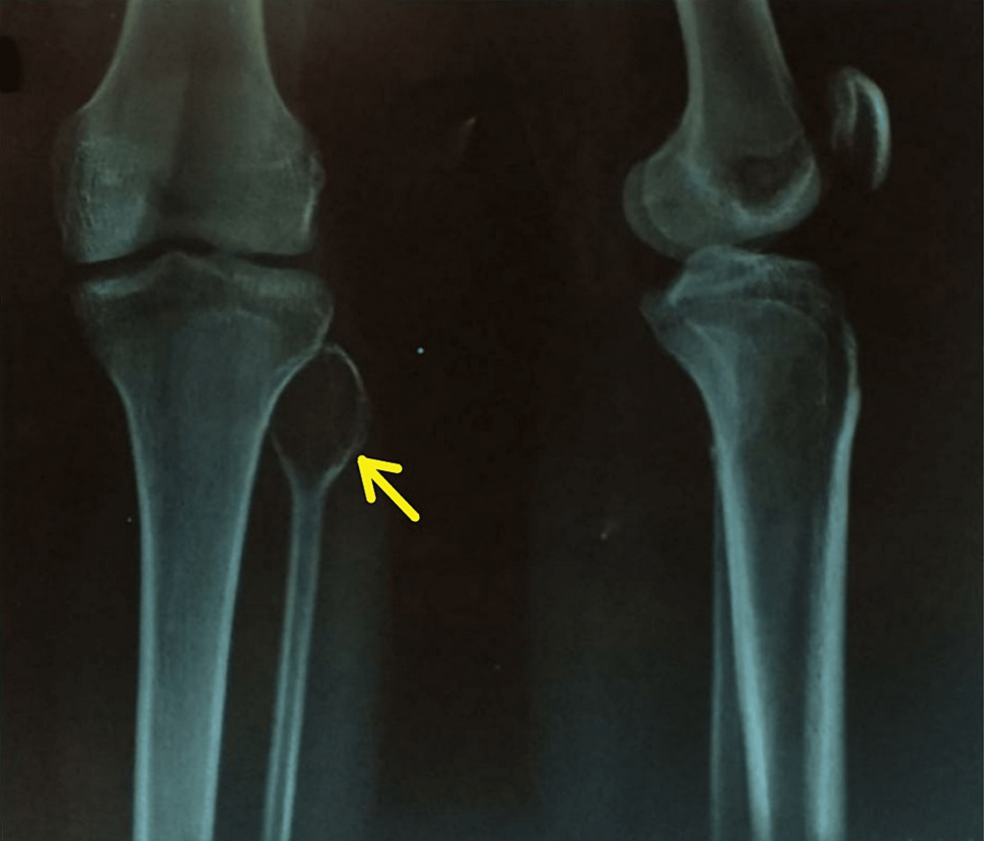
X-ray of the right knee with proximal one-third of the right tibia and fibula showing the radiolytic shadow and pencil-thin cortices of the head of the right fibula. The arrow marks the expansile ballooning lesion.

X-ray of the right knee along with proximal one-third of the right tibia and fibula (Figure 1) was performed and showed evidence of an expansile lesion of the fibular head, showing radiolytic shadow with pencil-thin cortices of the head of the fibula. No trabecular patterns were seen inside. This radiolucent area is reaching up to the neck of the fibula.

**Figure 2:**
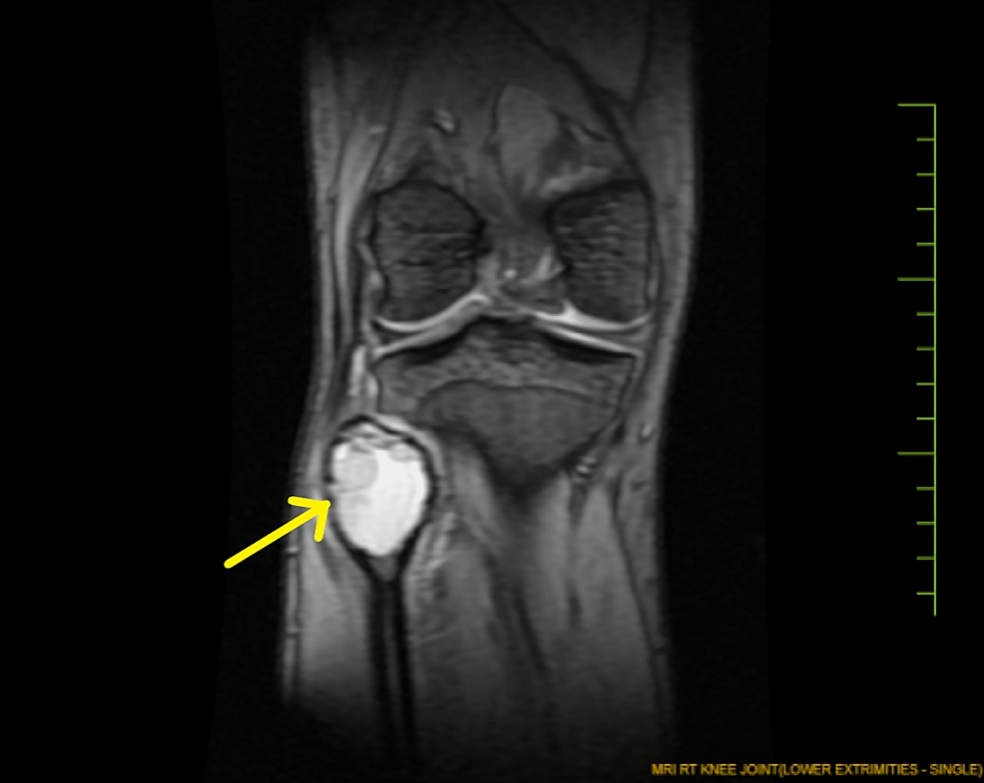
Magnetic resonance imaging of the right knee. The arrow marks the expansile cystic lesion involving the right fibular head. It shows the thinning of the cortex.

**Figure 3:**
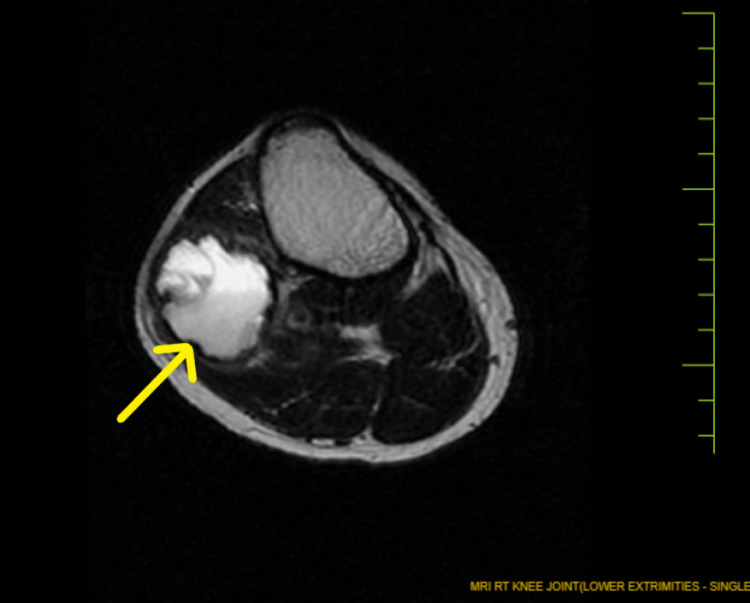
Transverse section of the magnetic resonance imaging of the right knee joint showing the cystic lesion marked by the arrow and the blood fluid level.

Magnetic resonance imaging (MRI) of the right knee (Figure 2 and Figure 3) suggests an aneurysmal bone cyst in the proximal right fibula. There is evidence of a well-defined expansile cystic lesion involving the metaphysis of the proximal end of the fibula with multiple septae and blood fluid levels within it causing thinning of the cortex. There is associated soft tissue edema. The lesion measures approximately 4.3 × 4 × 3.6 cm.

Intrasubstance T2-weighted image (T2WI)/proton density fat saturation (PDFATSAT)/short-tau inversion recovery (STIR) hyperintensity in the anterior cruciate ligament is suggestive of a partial tear.

Treatment

Based on the findings above, en bloc excision of the fibular head was done, taking care of the common peroneal nerve. The tumor was resected. The feeding vessels of the cyst were ligated. Antibiotic coverage was ensured. The patient was mobilized with a walker with partial weight-bearing. Vigorous quadriceps strengthening exercises were advised. The excised fibular head was subjected to histological examination.

The operative steps were as follows. A three-layered sterile dressing was done. The site of the operative procedure was painted with 10% povidone-iodine. Tourniquet was applied with pressure set up at 280 mm Hg. A curvilinear incision was given over the lateral aspect of the proximal femur, centering over the femoral head, extending up to the femoral shaft approximately 8-10 cm. The skin and subcutaneous tissue were incised and retracted. The lateral collateral ligament was dissected, elevated, and retracted. The common peroneal nerve was exposed carefully, tagged, and retracted, thus exposing the fibular head. The fibular head was exposed all through the way 360 degrees. The encapsulated tumor mass over the femoral head extending up to the femoral neck as well as the proximal fibular shaft was identified. In toto resection of the tumor, including the fibular head, neck, and 2.5 cm distal to the fibular neck, was performed. A thorough wash with normal saline was done, and 10% povidone-iodine was given. Anastomosis was achieved, the common peroneal nerve was untamed, and patency was noted and documented. Again, a thorough wash with normal saline was done, and 10% povidone-iodine was given. A layer-by-layer closure was done using vicryl 0 (skin and subcutaneous tissue, with grip ethyl on 2 0). Distal pulses (dorsalis pedis and posterior tibial artery) were noted, found satisfactory, and documented. The knee joint along with the proximal tibia was viewed under the C arm in anteroposterior (AP) and lateral views, which showed anatomical resection of the tumor over the fibular head and neck, which was preoperatively templated. Sterile dressing was applied. Above knee backslab was applied with 15-20 degrees flexion at the knee joint.

Histopathological examination

**Figure 4:**
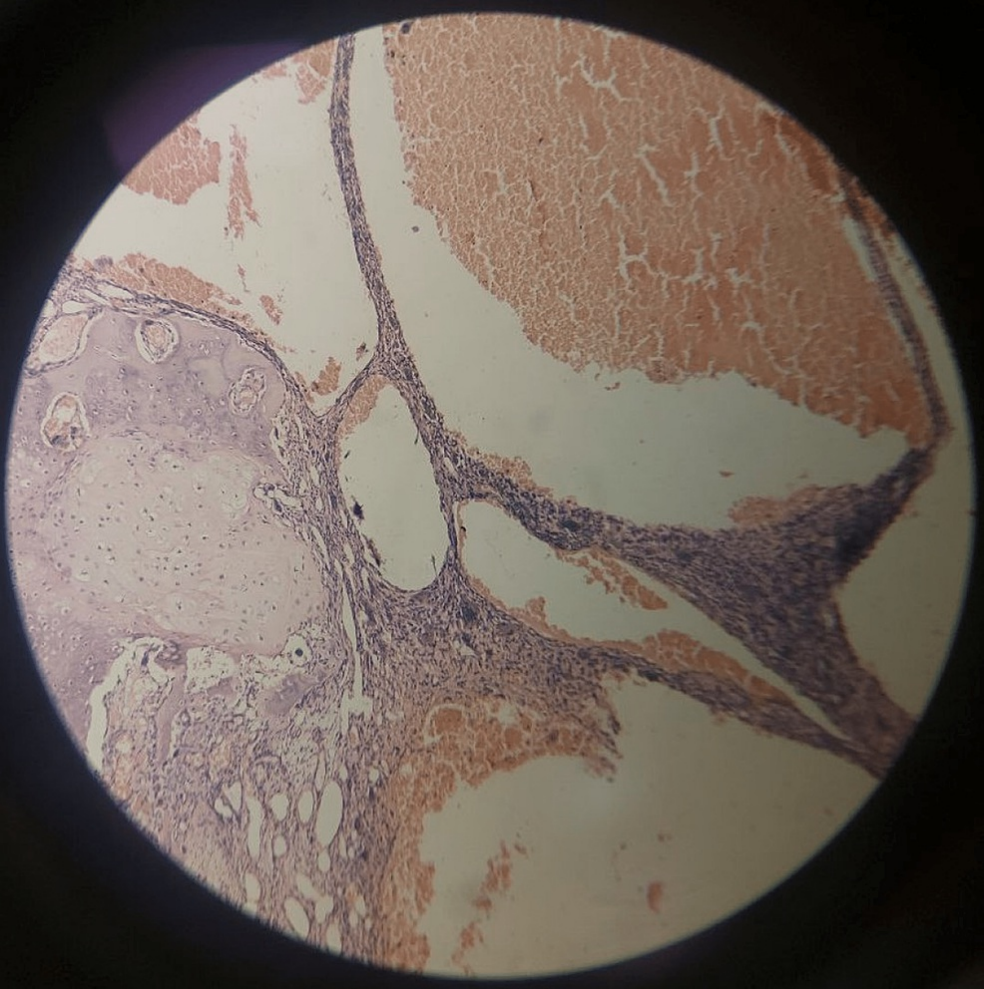
Photomicrograph of the excised cyst at low power field (20×) showing the bone (left), blood-filled cystic areas with giant cells, fibroblasts, and inflammatory cells lining the cyst.

**Figure 5:**
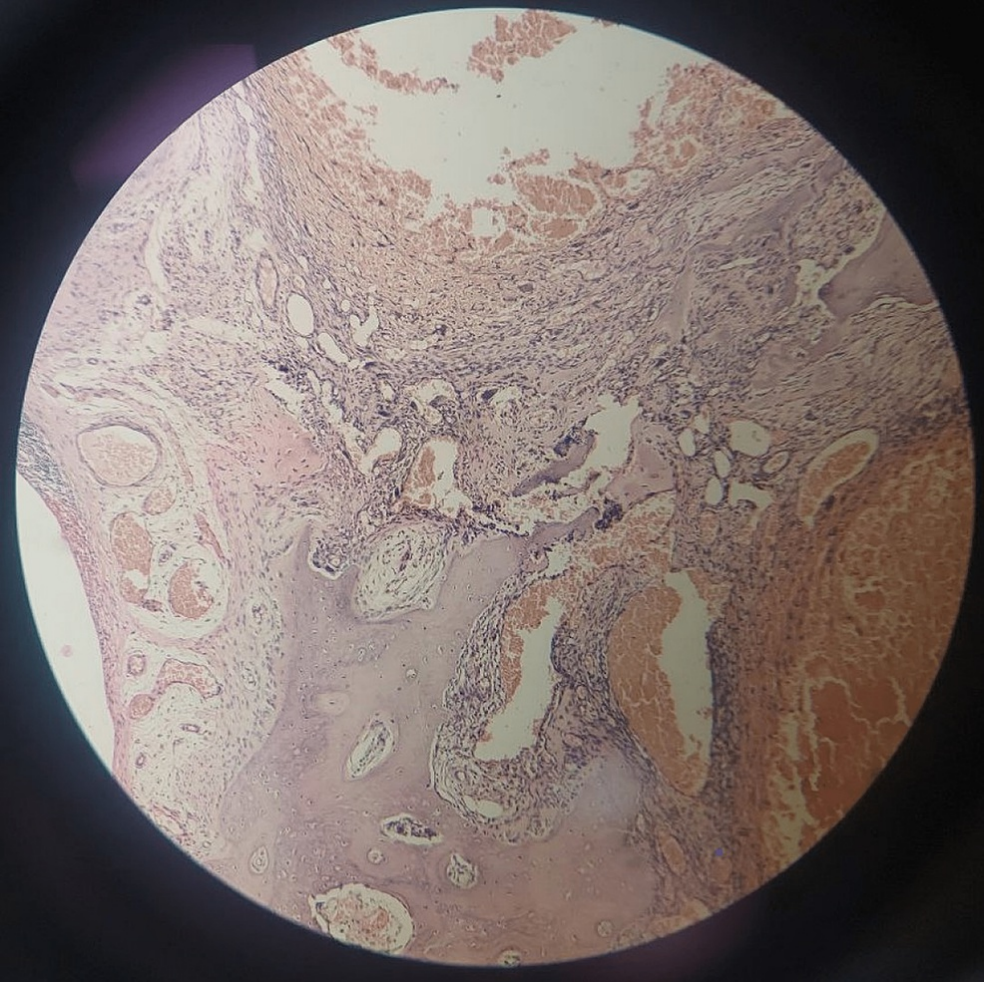
Photomicrograph of the excised cyst at low power field (20×) showing the bone, blood-filled cystic areas with giant cells, fibroblasts, and inflammatory cells lining the cyst.

**Figure 6:**
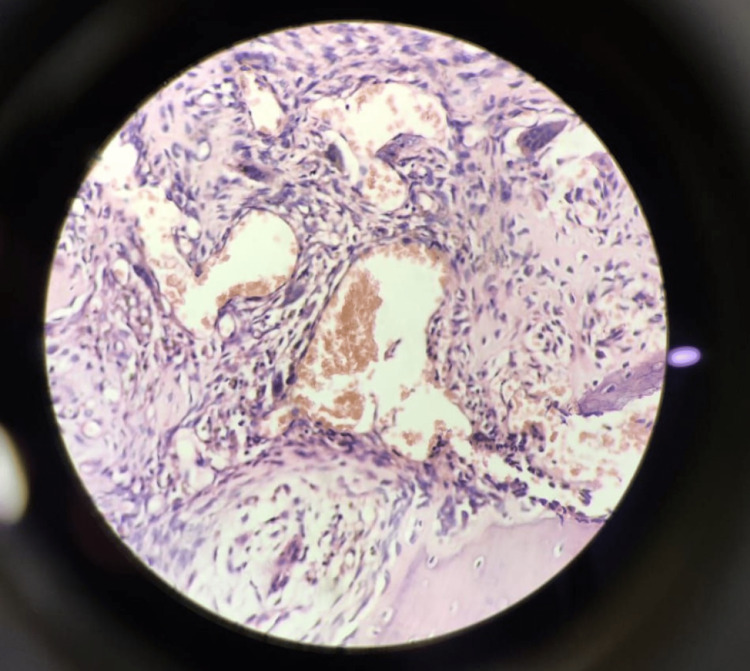
Photomicrograph of the excised cyst at high power field (40×) showing blood-filled cystic areas with giant cells, fibroblasts, and chronic inflammatory cells such as fibroblasts and plasma cells lining the cyst.

Histopathological examination of the excised aneurysmal bone cyst, when seen in low power field (20×) (Figure 4 and Figure 5), shows bone at one end and blood-filled cystic spaces lined by giant cells, fibroblasts, and chronic inflammatory cells such as lymphocytes and plasma cells. On high power field (40×) (Figure 6), blood-filled cystic spaces enclosed in fibro-osseous septa with supporting connective tissue bordering the vascular spaces were seen.

Diagnosis

**Figure 7:**
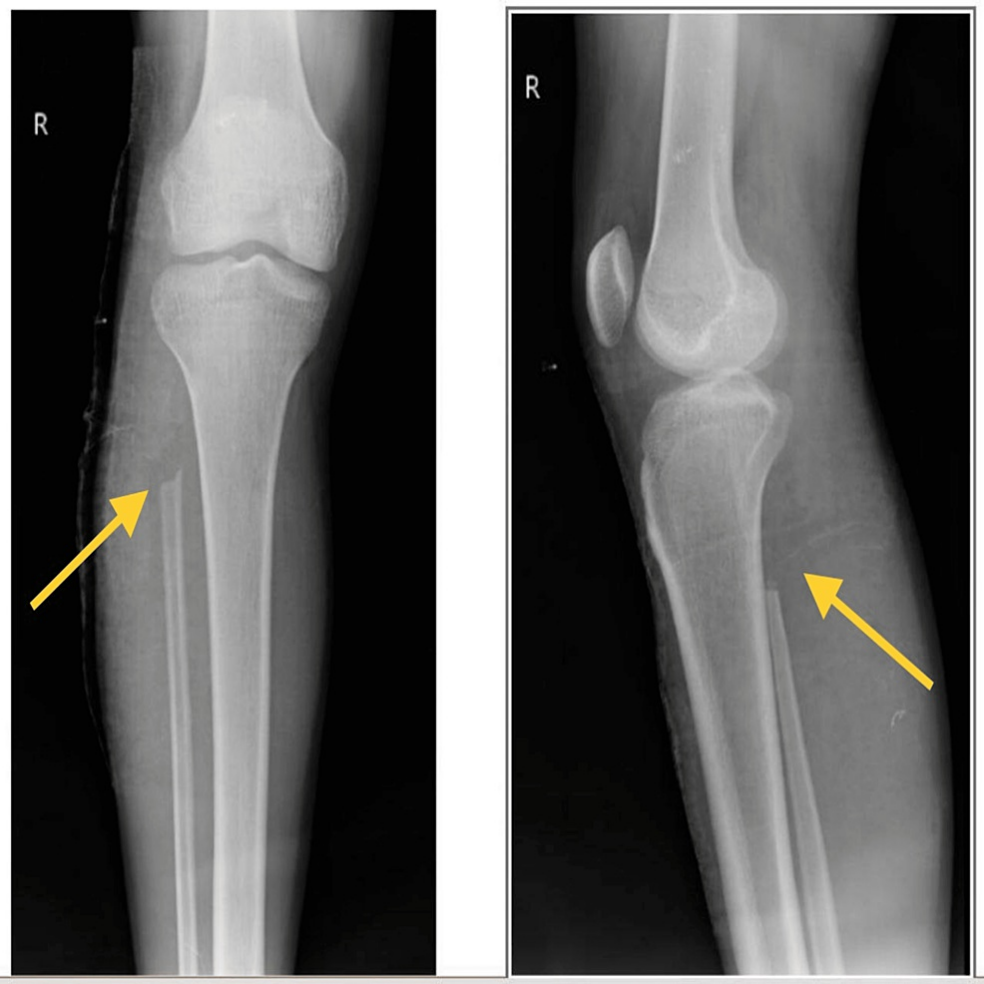
Postoperative X-ray (anteroposterior and lateral view) of the right knee joint with proximal tibia and fibula showing absent fibular head (arrows) as it was excised in the en bloc resection of the cyst.

The diagnosis was an aneurysmal bone cyst in the proximal aspect of the right fibula. Postoperative X-ray AP view of the right knee and proximal one-third of the tibia and fibula (Figure 7) shows the absence of the fibular head as it was excised.

**Figure 8:**
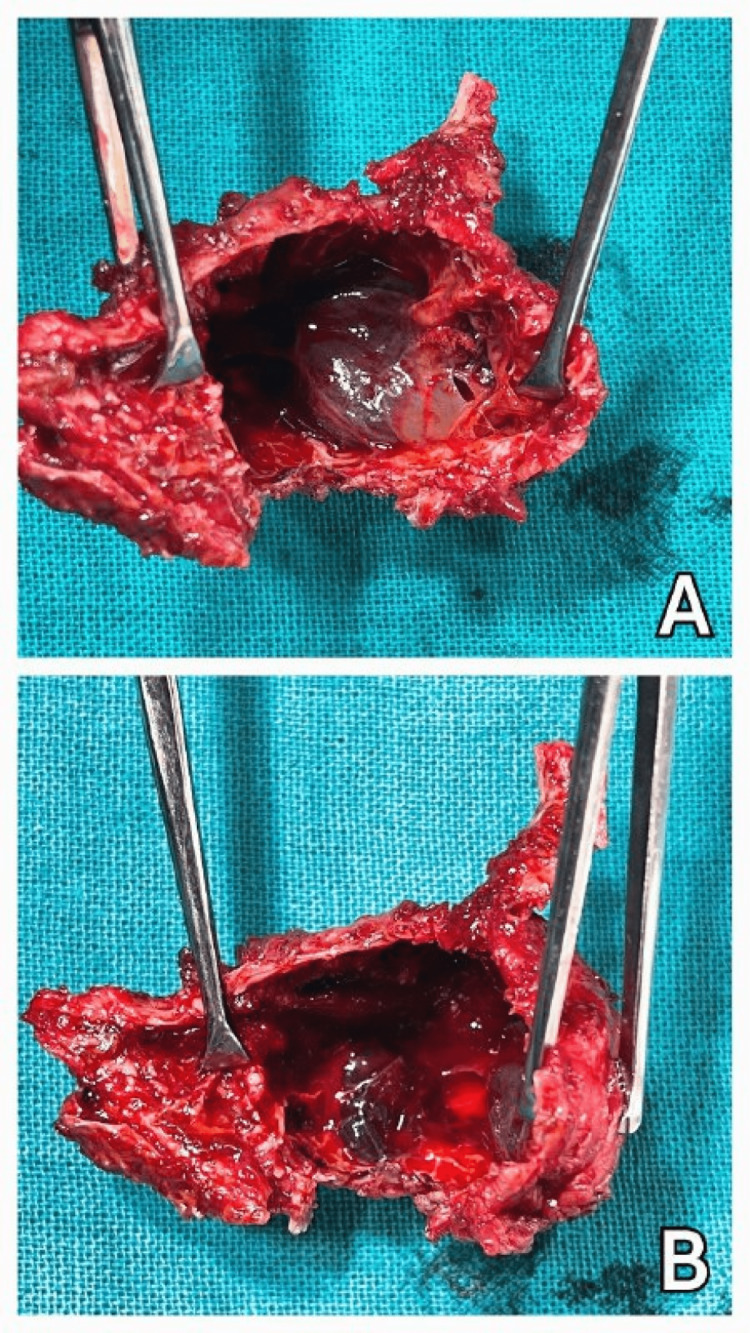
Thin shell of bone enclosing cystic blood-filled spaces (A and B).

**Figure 9:**
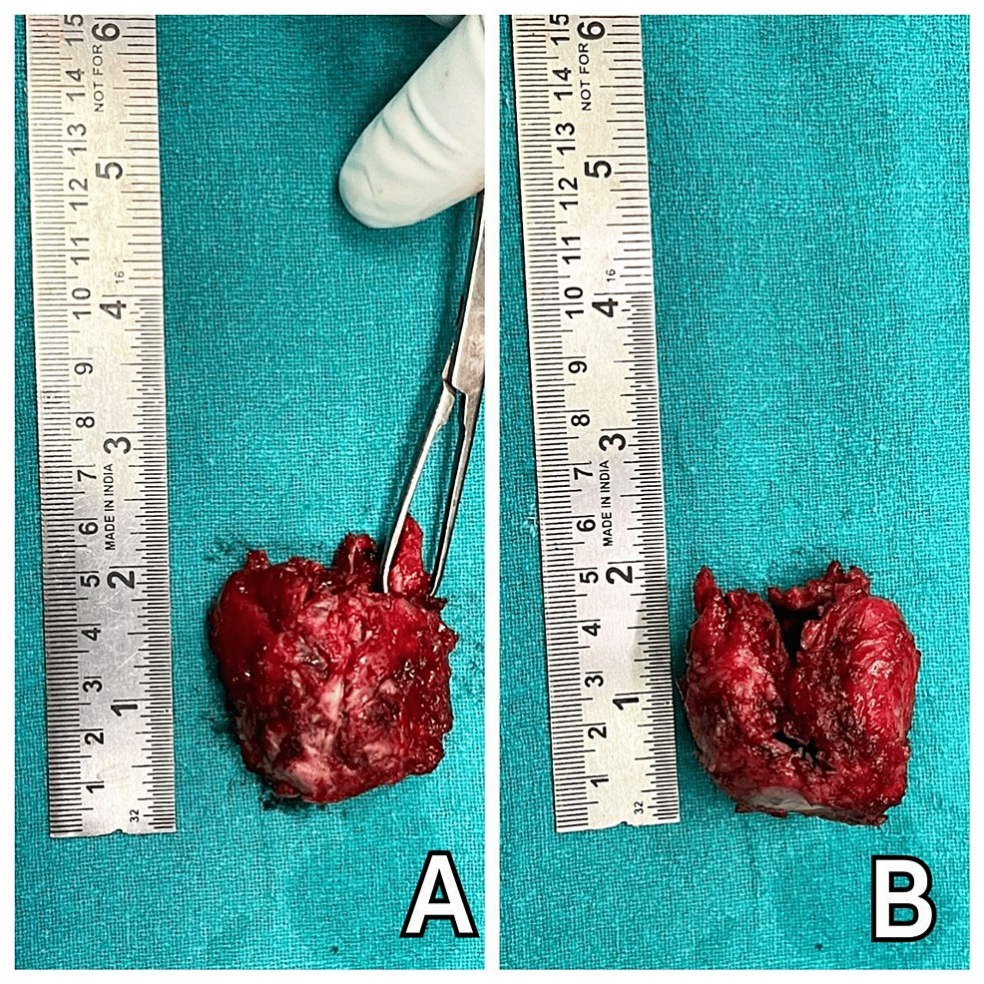
The excised cyst measuring 5 × 4.5 cm (A and B).

The excised specimen of the cyst (Figure 8 and Figure 9) shows a thin shell of bone enclosing cystic blood-filled spaces. The whole cancellous bone has been destroyed. It measured 5 × 4.5 cm in size.

## Discussion

Bone cysts are cystic, fluid-filled benign lesions of the metaphysis of long bones and vertebrae. It affects the population ranging from the pediatric age group to adolescents. It can be a unicameral or aneurysmal type of bone cyst. A unicameral cyst is a simple cystic lesion, whereas an aneurysmal cyst is a blood-filled cavity that is locally invasive and hemorrhagic at times [1-4]. An aneurysmal bone cyst (ABC) is of two types: primary or classic and secondary.

**Figure 10:**
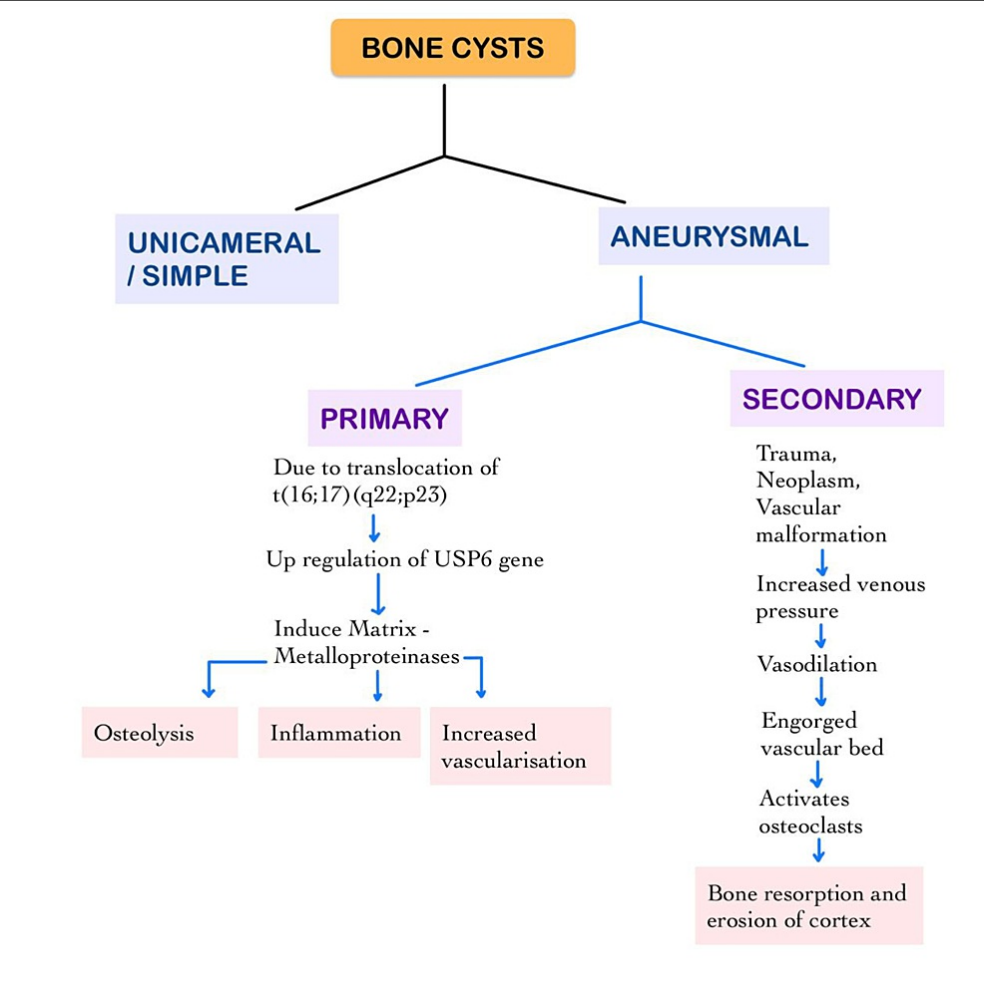
Types of bone cysts and pathogenesis of aneurysmal bone cysts.

Primary ABC does not have any pre-existing lesion, while secondary ABC may be preceded by any primary malignant tumor or metastasis [1,2,6]. ABC shows a female preponderance [3,4,6]. Etiologic factors for ABC include trauma, vascular malformations such as arteriovenous fistula or any abnormal circulatory pattern, and pre-existing neoplastic lesions such as chondroma and fibrous dysplasia [1,4,6-8]. The pathogenesis of aneurysmal bone cysts usually pertains to the abnormality in venous circulation that further on leads to increased venous pressure, vasodilatation, and thus distended vascular bed. This distended vascular bed additionally brings about the resorption of bone and the erosion of the cortex [1,4,6,7]. The primary variant is said to be linked with translocation-induced upregulation of ubiquitin-specific protease USP6, which causes osteoclastic activity (Figure 10) [7-9].

The clinical features of ABC include gradual onset of pain at the site, perceivable bony swelling, local temperature rise, decreased range of motion, and numerous pathological fractures (rarely found). Such uncertain symptoms may delay the diagnosis further. In the case of the involvement of any nerve, it may present with the above symptoms in combination with torticollis, scoliosis, and neurologic symptoms such as sensory impairment and paresis [1,3,4,6,7].

Radiological investigations such as X-rays, MRI, and CT scans are crucial to corroborate the diagnosis. X-ray findings do not confirm the diagnosis but can give evidence of cystic lesions that are eccentric, expansile, osteolytic, and trabeculated and have led to the loss of contour of bone [1,2]. MRI study shows a soap bubble appearance. It also indicates segmented, multiseptated, protractile areas of low signal intensity within and usually fluid levels. These fluid levels have a separation of serum that is non-dependent and of high signal intensity and red blood cells that of low signal intensity with deoxyhemoglobin. Besides, a lesion, if bordered by a low signal rim, implies a benign lesion; the rim’s intensity increases on T2-weighted MRI. Pathological fractures can also be visualized with surrounding soft tissue and osseous edema [1,3-7].

CT scans show extensile lesions accompanied by a narrow transitional zone with low-density (serum) and high-density (red blood cells) fluid separated from each other. Distended diploic spaces can also be seen [4,6].

Other investigations include scintigraphy with technetium-99m, manometry, and angiography (also used in embolizing the feeder vessels of ABC) [1,5,6].

The diagnosis can be confirmed on histopathological examination of the excised cyst [3,9,10]. Microscopic visualization shows hemosiderin-ladened cystic cavities with septa of the trabeculated bone and lacey osteoid tissue. Since no endothelium is seen, it confirms that no true blood vessel is present. Stromal constituents include fibroblasts, spindle cells, osteoid cells, multinucleate giant cells, and inflammatory cells. Abnormal mitosis should not be present. In the case of primary ABC, anastomosing fibrous walled channels are present that contain blood. Secondary ABC will have the features mentioned above, including those of the underlying cause [1,4,6].

The differential diagnosis includes giant cell tumor, telangiectatic osteosarcoma, chondroblastoma, and fibrous dysplasia [4,5,7].

Treatment options for ABC include a range of techniques based on the location of the cyst, primary pathology, and the patient’s condition. Some of the treatment options are surgical and non-surgical interventions. The surgical intervention, being the gold standard, includes techniques such as en bloc or total resection, partial excision, or intralesional curettage with adjunct therapy. Intralesional excision can be further followed by filling the bone with any bone graft or cement. However, total excision has lower recurrence rates but is not usually done [1-4,7]. While performing surgery, the peroneal nerve and the lateral structures of the knee must be taken care of [10]. Non-surgical or adjunct therapy options are sought when there is a need to conserve the anatomy and performance of the structure. It includes the following modalities: percutaneous sclerotherapy, cryotherapy, high-speed burr, argon beam coagulation, phenol, selective artery embolization (SAE) (more preferred nowadays), endoscopic curettage, concentrated bone marrow injection, curopsy, percutaneous curettage, cyst aspiration, and polidocanol sclerotherapy. Radiotherapy can also be used [1-4,6-11]. Rehabilitation for the fractures should be recommended. External fixation and plate osteogenesis are preferred for fracture correction [2,10].

Monoclonal antibodies such as denosumab have also been under research for treating ABC recently [2,4].

## Conclusions

Bone cysts are tumor-like pathology of bone occurring in the younger population. It can occur at sites such as metaphyseal ends of long bones, flat bones, and vertebrae. They are of two types: simple/unicameral and aneurysmal bone cysts. Aneurysmal bone cysts are further classified as primary and secondary ABC. Investigations help in identifying and confirming the diagnosis. Treatment modalities are surgical and non-surgical. Surgical methods include total/en bloc resection, bone grafting, and partial resection. Non-surgical methods include radiotherapy, cryotherapy, and curopsy.
